# Hypoxia, endoplasmic reticulum stress and chemoresistance: dangerous liaisons

**DOI:** 10.1186/s13046-020-01824-3

**Published:** 2021-01-11

**Authors:** Muhlis Akman, Dimas Carolina Belisario, Iris Chiara Salaroglio, Joanna Kopecka, Massimo Donadelli, Enrico De Smaele, Chiara Riganti

**Affiliations:** 1 Department of Oncology, University of Torino, via Santena 5/bis, 10126 Torino, Italy; 2 Department of Neurosciences, Biomedicine and Movement Sciences, Section of Biochemistry, University of Verona, Verona, Italy; 3 Department of Experimental Medicine, Sapienza University of Roma, Roma, Italy

**Keywords:** Hypoxia, Hypoxia-inducible factor-1α, Endoplasmic reticulum stress, Unfolded protein response, Chemoresistance

## Abstract

Solid tumors often grow in a micro-environment characterized by < 2% O_2_ tension. This condition, together with the aberrant activation of specific oncogenic patwhays, increases the amount and activity of the hypoxia-inducible factor-1α (HIF-1α), a transcription factor that controls up to 200 genes involved in neoangiogenesis, metabolic rewiring, invasion and drug resistance. Hypoxia also induces endoplasmic reticulum (ER) stress, a condition that triggers cell death, if cells are irreversibly damaged, or cell survival, if the stress is mild.

Hypoxia and chronic ER stress both induce chemoresistance. In this review we discuss the multiple and interconnected circuitries that link hypoxic environment, chronic ER stress and chemoresistance. We suggest that hypoxia and ER stress train and select the cells more adapted to survive in unfavorable conditions, by activating pleiotropic mechanisms including apoptosis inhibition, metabolic rewiring, anti-oxidant defences, drugs efflux. This adaptative process unequivocally expands clones that acquire resistance to chemotherapy.

We believe that pharmacological inhibitors of HIF-1α and modulators of ER stress, although characterized by low specificty and anti-cancer efficacy when used as single agents, may be repurposed as chemosensitizers against hypoxic and chemorefractory tumors in the next future.

## Background: the impact of hypoxia on cancer and its microenvironment

Cancer growth is supported by the continuous interaction between transformed and non-transformed cells, including cancer-associated fibroblasts, endothelial cells and immune-infiltrating cells that constitute the so-called tumor microenvironment (TME).

Within TME, cancer cells are subjected to multiple stresses caused by shortage of oxygen (O_2_) and nutrients, chronic inflammation, damages induced by immune cells or exogenous factors, such as chemotherapy and radiotherapy. O_2_ shortage is the most common condition that tumors must face [1]. The highest O_2_ concentration (13.2%) is measured in arterial blood and is considered as normoxia [2]. Under physiological conditions, the O_2_ concentration in human tissues varies between 1 and 11%. Most solid tumors grow under hypoxic conditions, i.e. below 2% O_2_ [3, 4].

When facing hypoxic conditions, cancer cells can undergo two processes: slowing their progression and ending-up in necrosis/apoptosis, or adapting to the unfavorable conditions. This adaptation expands more aggressive clones that become predominant in the tumor heterogeneous population [5]. This process is mainly orchestrated by hypoxia-inducible factors (HIFs), i.e. specific hypoxia sensors that support cell survival by inducing compensatory angiogenesis, extracellular matrix (ECM) remodelling, metabolic shift and immune-suppression [6–10]. HIF proteins are dimers consisting of an unstable, O_2_-sensitive α subunit and a costitutively expressed, O_2_-insensitive β subunit. Three HIFɑ proteins - HIF-1ɑ, HIF-2ɑ and HIF-3ɑ, each with multiple splicing variants – and one HIFβ protein were identified in higher organisms [11]. Although HIF-1ɑ is ubiquitary and HIF-2ɑ prevails in heart and liver, both of them are expressed in many cancer types and have overlapping functions. Little is known on HIF-3ɑ, whose study is complicated by the multiple variants of this protein present in humans [12].

Under normoxia, the labile ɑ subunits are hydroxylated by the prolyl hydroxylase dioxygenase (PHD) enzymes [13], which generate a binding site for the von Hippel Lindau tumor suppressor protein (pVHL). Following the pVHL binding, HIFɑ is poly-ubiquitinated and then degraded by the proteasome [14, 15]. PHD is an O_2_-dependent enzyme: under hypoxic conditions, HIFɑ is not primed for ubiquitination, but it accumulates and binds to its β subunit, and is free to translocate into the nucleus [16, 17]. Besides hypoxia, HIF-1ɑ stabilization may be promoted by oncogenic activation of pro-survival pathways such as Ras/PI3K/Akt/mTOR [18], mutations in key oncosuppressor genes such as TP53 [19] or BRCA1/PTEN axis [20], metabolites paracrinely released in the TME such as glutamate [21]. Similarly, the intracellular accumulation of reactive oxygene species (ROS) inactivates PHD, increasing HIF-1ɑ within tumors [11].

Specific miRNAs stabilize HIF-1ɑ [22] and are involved in the hypoxia-mediated modulation of angiogenesis, apoptosis, proliferation, metastasis and chemoresistance [23]. For instance, the increase in HIF-1α is associated to the up-regulation of miR-155, miR-10b, miR-372, miR-373, miR-210 and miR-519c, and to the down-regulation of miR-17-92, miR-20b, miR-200b and miR-199 [24].

HIF proteins induce up to 200 genes that promote adaptation to hypoxia [6, 11], involved in proliferation, glucose metabolism and angiogenesis in many different cancer types [25–27]. Among them, there are glucose transporter 1 (GLUT1), which increases the glucose uptake fuelling the anaerobic glycolysis, and vascular endothelial growth factor (VEGF), a well-known angiogenic factor [28]. HIF-1α is a strong inducer of other glycolytic enzymes that contribute to an increased rate of anaerobic glycolysis, coupled with a lower rate of tricarboxylic acid (TCA) cycle. This phenotype is typical of several solid cancers addicted to glucose as the main fuel source [29]. In addition, HIF-1ɑ down-regulates oxidative phosphorylation (OXPHOS) by inducing the Hes-related family BHLH transcription factor with YRPW motif (HEY) repressor [30], decreases the expression of carnitine palmitoyl transferase 1A (CPT1A), the limiting-enzyme of fatty acid β-oxidation (FAO) [31] and inhibits the electron transport chain (ETC) [32]. As a result, both anaplerotic pathways of TCA cycle and ETC are limited. The main benefit derived from the inhibition of mitochondrial metabolism is the decreased level of ROS and the prevention of cytotoxic oxidative stress [33].

Both HIF-1ɑ and HIF-2ɑ promote a more invasive phenotype [33], by activating the epithelial-mesenchymal transition (EMT) program [34–36] or cooperating with other strong pro-invasive factors such as Met receptor and soluble hepatocyte growth factor (HGF) [37], or VEGF receptor (VEGFR)/VEGF [38]. Notably, in triple negative breast cancer (TNBC) HIF-1ɑ stimulates the relase of the typical pro-inflammatory cytokine IL-1β that induces a metastatic attitude in both tumor cells and cancer associated fibroblasts (CAFs), creating a TME that strongly favors metastatization [39]. In addition, HIF-1ɑ also promotes a tumor-tolerant environment, reducing the infiltration of CD4^+^ and CD8^+^ T-lymphocytes and tumor-associated macrophages (TAMs) [40], increasing the differentiation of T-lymphocytes into T-helper 17 (TH17) cells [41] and modulating TAM polarization [42]. Overall, the hypoxia-driven reshaping of immune-environment is associated to increased tumor progression, particularly in the early stages [43].

Given the pleiotropic functions regulated, HIF-1ɑ expression has been used as a stratification factor. Specific signatures of HIF-1ɑ-target genes have a negative prognostic significance in hepatocellular carcinoma (HCC) [44]. Similarly, a meta-analysis focused on the prognostic role of HIF-2ɑ demonstrated that a higher expression of this transcription factor is associated with a worst overall survival, metastasis-free and progression-free survival in melanoma, breast and lung cancer [45].

Hypoxia and nutrients shortage are two classical conditions inducing endoplasmic reticulum (ER) stress, a perturbation of the ER homeostasis that determines the accumulation of unfolded or misfolded proteins within ER lumen. As hypoxia does, also ER stress may evolve into two opposite situations: the activation of pro-apoptotic program, with consequent cell death in case of acute ER stress, or the activation of pro-survival responses, in case of chronic and mild ER stress [46]. In a strict parallelism with hypoxia, also ER stress determines the selection of tumor populations with a multistress-resistant phenotype, i.e. able to survive in the presence of unfavorable and changing conditions, such as O_2_ and nutrients shortage, or exposure to chemotherapeutic drugs [47]. Solid tumors experiment continuous changes in O_2_ and nutrients supply. Indeed, if the hypoxia-induced neo-angiogenesis provides O_2_ and nutrients, the neo-vessels formed easily collapse under the pressure of the growing tumor mass. This situation implies that repeated cycles of hypoxia and re-oxygenation occur within the tumor, and that normoxic and hypoxic areas coexist within the tumor bulk. The poorly organized vasculature also determines an irregular delivery of chemotherapeutic drugs. Tumors are heterogeneous masses composed by hypoxic niches, characterized by severe shortage of O_2_ and nutrients, and stronger ER stressing conditions, and well-oxygenated areas, less subjected to ER stress [48, 49]. In this review, we will discuss how the adaptation to hypoxia and to the hypoxia-induced ER stress contribute to the expansion of aggressive tumor clones and to the acquisition of chemoresistance, by triggering pro-survival/anti-apoptotic responses. A better knowledge of the cross-talks betweeen hypoxia and ER stress may open new therapeutic opportunities effective against tumors refractory to classical chemotherapy, by targeting HIF activation and modulating the response to ER stress.

## The variable response to ER stress in cancer cells

In eukaryotic cells, protein folding and maturation are handled by ER, where nascent polypeptide chains synthesized by ribosomes are folded and undergo post-translational modifications, such as disulfide bonds formation and glycosylation. Synthesized proteins must pass the ER-associated protein degradation/ER-quality control (ERAD/ERQC) system, constituted by ER-associated protein complexes that deliver the properly folded proteins to their final destination, the unfolded/misfolded proteins to ubiquitination and degradation via proteasomes or autophagosomes/lysosomes [50, 51]. ERAD/ERQC proteins are often over-expressed in cancers [52], as part of an adaptive response that help cancer cells to survive in unfavorable environments. Upon glucose deprivation, oxidative stress, aging or hypoxia, ERAD/ERQC system can be overwhelmed. Unfolded proteins accumulate within the ER lumen, triggering the so-called unfolded protein response (UPR) that results in cell death or ER stress compensation and consequent survival [46, 53, 54].

When unfolded proteins accumulate, ER-resident chaperones such as glucose-regulated protein 78 (GRP78) increases the folding capacity or proceeds to the clearance of unfolded proteins via ERAD machinery. In parallel, protein translation is reduced, to limit the burden of unfolded proteins accumulated within the ER lumen [55]. If both options fail, cells initiate apoptosis [54]. Inositol-requiring enzyme-1α (IRE1α), activating transcription factor-6 (ATF6) and protein kinase R-like endoplasmic reticulum kinase (PERK) are ER transmembrane proteins that act as ER-stress sensors and UPR effectors [46, 53, 54], and are activated by GRP78 [56].

IRE1α activates X-box-binding protein 1 (XBP-1) that up-regulates genes increasing proteins folding, ERAD machinery and ER biogenesis [57, 58], or – if the ER stress persists – up-regulates genes involved in apoptosis, such as c-Jun N-terminal kinase 1 (JNK1) [56].

ATF6 controls the second arm of UPR. It increases the transcription of chaperones and ERAD pathway members, as well as GRP78, generating a feed-forward loop which buffers ER stress [55].

The third sensor of UPR is PERK that - by phosphorylating on serine the eukariotic initiating factor 2α (eIF2α) - reduces the mRNA translation rate, attenuating the accumulation of misfolded proteins within ER [58, 59]. At the same time, PERK increases the translation of the activating transcription factor-4 (ATF4) that induces the transcription of chaperons, anti-oxidant enzymes and enzymes involved in aminoacid metabolism, favoring resistance to oxidative stress and drugs [56]. ATF4 indeed may promote the transcription of CCAAT/enhancer-binding protein homologous protein (CHOP) that induces apoptosis in case of prolonged stress [46, 53, 54], but also protective autophagy [60].

Multiple cross-talks between the three arms of ER stress exist. For instance, ATF6 also enhances IRE1α/XBP-1 axis [61]. Together, they promote the degradation of misfolded proteins during embryonic [62] and cancer development [63]. Also PERK activates ATF6, by favoring its synthesis and translocation from ER to Golgi [64]. Interestingly, as observed for HIF-1α [22, 24], miRNAs act both as controllers of expression of UPR proteins and modulators of the UPR response [65]. Only in one case, miRNA expression changes in the same direction under hypoxic conditions and ER stress: mir-17, which is down-regulated by HIF-1α [24], is also reduced during ER stress. Such decrease, favored by the activation of IRE1α, promotes the switch from an adaptive UPR response to a pro-apoptotic response [65]. This parallelism suggests that specific miRNAs may contribute to the effects that both HIF-1α and ER stress have on the cell fate. These miRNAs may be part of the same molecular circuitries, linking the hypoxic-induced ER stress to the ER stress-induced cell death or survival. However, further analyses are needed to unveil if other miRNAs have the same modulation in case of hypoxia and ER stress, or if hypoxia-dependent and UPR-dependent miRNAs belong to two different and independent sets.

Overall, the redundancy of pathways facing ER stress, the versatility of the response observed and the circuitries influenced in the same way by hypoxia and ER stress suggest that UPR is a crucial and finely regulated mechanism controlling the cell fate in response to pleiotropic unfavorable conditions.

## Hypoxia and ER stress: a double-liaison selecting aggressive and resilient clones

Hypoxia is often a cause of ER stress, for at least two reasons: first, specific processes physiologically occurring within ER are altered in hypoxic conditions; second, HIF-1α modulates the expression and activity of ER stress sensors.

Many post-translational modifications are O_2_-dependent, e.g. the oxidation and isomerization of cysteine thiol groups that form disulfide bonds, or the hydroxylation of collagen on proline residues. In hypoxia, proteins are regularly synthesized but their folding is impaired and induces ER stress [66, 67]. Moreover, under hypoxic conditions, several splicing variants of common proteins are produced [68]: these variants may be sensed as abnormally folded proteins by the ERAD/ERQC apparatus, triggering a UPR response. Finally, the altered OXPHOS metabolism that is often observed under hypoxia generates ROS, which oxidize mitochondrial proteins and/or impact on their folding. This triggers a mitochondrial UPR (mUPR) that is additive to the ER-dependent UPR [69, 70].

On the other hand, HIF-1α controls the activity and expression of UPR sensors. HIF-1α transcriptionally up-regulates VEGF, which activates phospholipase C (PLC) and inositol-3-phosphate (IP3)-dependent calcium release: the oscillations in calcium levels trigger the activation of UPR arms [71]. Although this mechanism was reported in human endothelial cells, a similar cascade of events may occur in hypoxic cancer cells, rich of VEGFR and autocrinely producing VEGF. In addition, hypoxia increases GRP78 expression via extracellular signal-regulated kinase (ERK) and protein kinase C (PKC) [72], thus activating the *primum movens* of the three UPR arms. One mechanism explaining the hypoxia-driven increase of GRP78 relies on the cell migration-inducing and hyaluronan-binding  protein (CEMIP), an ER-residing protein that is overexpressed in several solid cancers in response to hypoxia. CEMIP transcriptionally up-regulates GRP78 and binds it in the ER: the CEMIT/GRP78 complex increases glucose uptake, prevents apoptosis and promotes cell migration by raising the intracellular calcium and the activity of PKCα [73], thus producing a better adaptation to hypoxia.

The final results of the hypoxia-induced UPR are variable, and depend on the degree and duration of hypoxia. For instance, while HIF-1α and HIF-2α are activated starting from < 3% O_2_, when the O_2_ tension is reduced to 0.1% the mammalian target of rapamycin (mTOR) is progressively inhibited and eIF2α is phosphorylated: these two events reduce the global translation of proteins [74].

The key role of PERK/eIF2α axis in pro-survival response during hypoxia has been demonstrated by the fact that cancer cells with an intact PERK/eIF2α axis are more tolerant to hypoxia and more tumorigenic [75]. These findings suggest that PERK activity makes cancer cells more resilient and aggressive. Notably, the activation of PERK is HIF-1α-dependent and is stronger after normoxia-hypoxia cycles, a condition often occurring within tumor bulks where the vasculature is irregular [56]. The acquisition of hypoxia-tolerance following PERK activation is mainly mediated by the downstream effector ATF4. Indeed, tumors with defective PERK/eIF2α/ATF4 axis have apoptotic areas overlapping with hypoxic areas [75], suggesting that a functioning PERK/eIF2α/ATF4 is required to limit the hypoxia-induced damages. Moreover PERK/eIF2α signalling buffers the increasing ROS levels observed in cyclic hypoxia, by increasing GSH and autophagy. These events have improved the survival of glioblastoma cells in response to the oxidative damages induced by oscillating hypoxia or radiotherapy [76, 77]. In human cervix cancer, the hypoxic induction of PERK/eIF2α/ATF4 signalling up-regulates the pro-metastatic protein lysosomal-associated membrane protein 3 (LAMP3) [78], linking hypoxic activation of PERK to another aggressive behavior of cancer cells.

IRE1α/XBP-1 and ATF6 are also up-regulated during hypoxia, although the biological meaning of their activation has been less studied. XBP-1 transcription and splicing are higher in hypoxic tumors [79]. Like PERK, also IRE1α modulates autophagy in response to hypoxia [80], mounting a cytoprotective response. The use of selective inhibitors of PERK and IRE1α, however, clarified that the blocking of IRE1α and XBP-1 splicing did not reduce cell proliferation during hypoxia, contrarily to the inhibition of PERK [81]. These observations suggest a predominant role of PERK/eIF2α axis over IRE1α/XBP-1 axis in hypoxia tolerance and tumor growth.

If it is well documented that hypoxia induces ER stress, it is also true that ER stress sensors increase HIF-1α activity and hypoxia-related events. Indeed, UPR signalling potentiates the response to hypoxia by phosphorylating and activating HIF-1α, and by stabilizing VEGF protein via the up-regulation of multiple chaperones [82]. Also ATF4 and IRE1α are transcriptional activators of VEGF in hypoxic cells, where they cooperate with HIF-1α [83]. Such crosstalk between hypoxia and UPR is not limited to specific genes, but it is part of a more generalized response: in hypoxic TNBC, XBP-1 enhances the transcription of HIF-1α-target genes by directly interacting with HIF-1α and recruiting the RNA polymerase II on specific promoters. This XBP1-HIF-1α-dependent signature is associated with tumor aggressiveness and poor prognosis [84].

Sometimes UPR- and hypoxia-driven responses do not act in the same direction. Depending on the temporal order of the events (i.e. UPR preceeding hypoxia or hypoxia determining UPR), on the severity and duration of hypoxia or ER stress, there can be contrasting responses. For instance, upon strong ER stress, ATF4, activated by p38 mitogen activated kinase (MAPK), down-regulates apelin, an anti-apoptotic protein, which is up-regulated by HIF-1α [85]: this is a typical case where the induction of strong ER stress before the up-regulation of HIF-1α induces cell death, preventing the typical anti-apoptotic response mediated by HIF-1α. In a complementary situation, if hypoxia triggers a strong ER stress, ROS/p38 MAPK axis, activated by HIF-1α, elicits a pro-apoptotic response mediated by ATF4 [86], instead of a proliferative response as expected upon the usual activation of MAPK proteins. Similarly, in breast cancer cells HIF-1α induces immediately VEGF that favors neo-angiogenesis and cell survival, but prolonged hypoxia activates the IREα/XBP-1 axis that induces miR-153, a negative regulator of HIF-1α [87]. Once the acute hypoxic insult is over and a chronic hypoxic environment persists, cells switch off the pro-survival programs driven by HIF-1α and switch on pro-apoptotic programs. A two-phases response is observed in prostate cancer (PC) and breast cancer cells, as well as in osteosarcoma exposed to 1% O_2_ tension: after an initial up-regulation of HIF-1α, the protein is gradually reduced notwithstanding the persistence of hypoxic conditions. Indeed, after 2–3 h of hypoxia, a strong UPR is mounted, characterized by the activation of PERK that destabilizes HIF-1α mRNA by disrupting its interaction with the stabilizing protein YB-1 [88]. In this case, the pro-survival programs induced by HIF-1α are attenuated and the pro-apoptotic programs controlled by UPR determines cell death.

Beside the duration and extent of hypoxia or ER stress, the presence of specific oncogenic pathways is another factor that may direct hypoxia- and UPR-dependent programs towards antagonistic directions. For example, in colon cancer the hyper-activated WNT/β-catenin axis disrupts the interaction between XBP-1 and HIF-1α, attenuating the transcriptional activity of HIF-1α and decreasing the adaptation to hypoxia [89]. This response is in contrast with what observed in TNBC, where – in the absence of non-oncogenically active β-catenin -, XBP-1 and HIF-1α cooperate in up-regulating a common set of target genes [84].

These prototypical examples suggest that HIF-1α- and UPR-driven pathways can be linked by synergistic or antagonistic cross-talks, depending on duration, degree and timing of hypoxia and ER stress. Adaptation to hypoxia and UPR are dynamic processes. Hence, static measures cannot be conclusive because they do not follow the evolution of hypoxic insults and the cellular compensatory responses. Time-lapse approaches provide significantly more information on the temporal and molecular hierarchy of HIF-1α- and UPR-dependent events, unveiling the cause-effect relations existing in each situation.

**Fig. 1:**
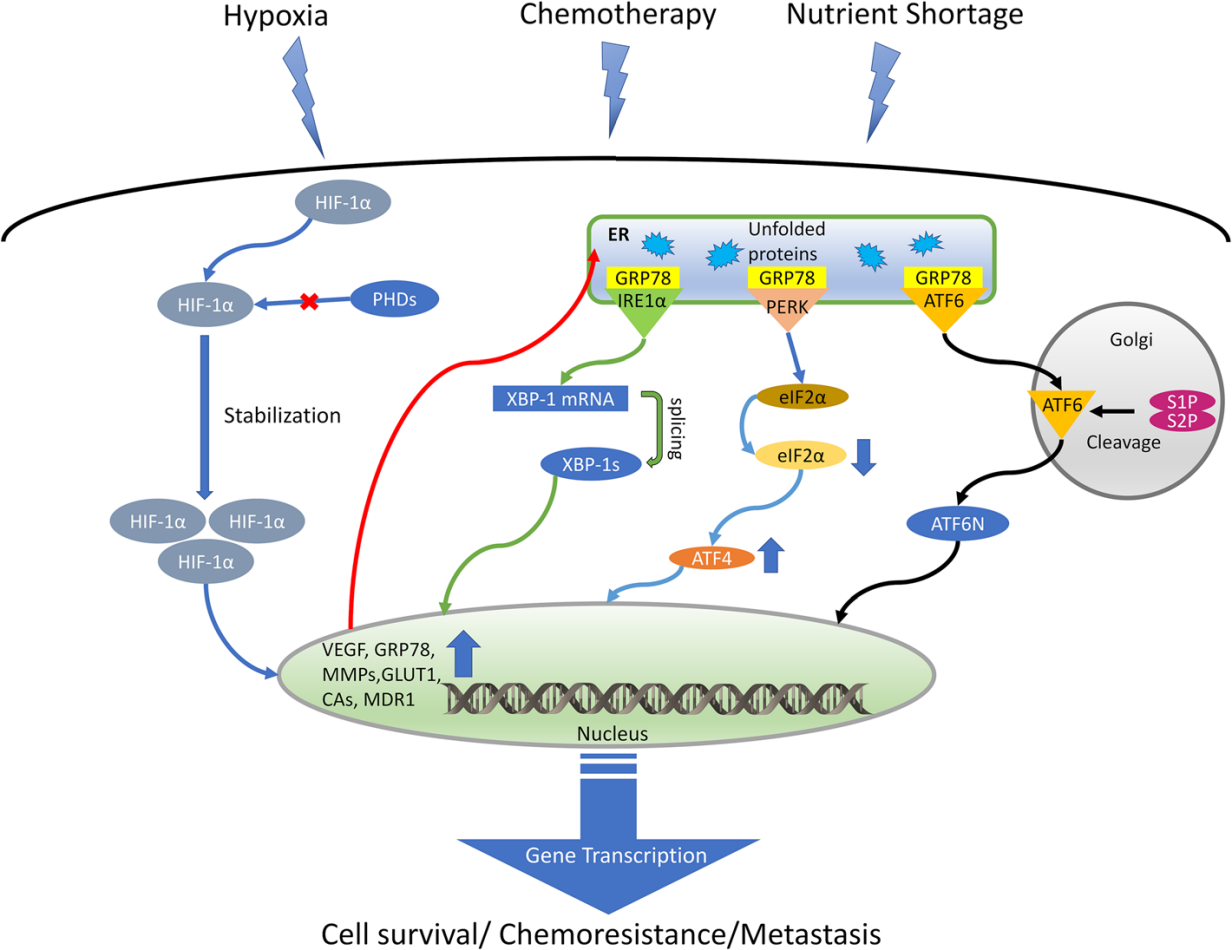
Hypoxia and ER stress select aggressive tumor clones. Hypoxia increases the stabilization of the hypoxia-inducible factor-1α (HIF-1α), by preventing its degradation operated by prolyl hydroxylase dioxygenase (PHDs) enzymes. Together with chemotherapy and nutrient shortage, hypoxia is also a strong inducer of ER stress. The increased burden of unfolded proteins is sensed by the glucose-regulated protein 78 (GRP78), which is also a HIF-1α-target gene. The GRP78 downstream effectors – namely inositol-requiring enzyme-1α (IRE1α), activating transcription factor-6 (ATF6) and protein kinase R-like endoplasmic reticulum kinase (PERK) – are activated. IRE1α induces the splicing (s) of X-box-binding protein 1 (XBP-1) into its active form; PERK phosphorylates the eukariotic initiating factor 2α (eIF2α) that increases the translation of activating transcription factor-4 (ATF4); ATF6 is cleaved by the Golgi site-1/site-2 proteases (S1P, S2P) into its nuclear (N) translocated form. XBP-1 s, ATF4 and ATF6N cooperate with HIF-1α in increasing the transcription of genes involved in neo-angiogenesis (vascular endothelial growth factor, VEGF), invasion (matrix metalloproteases, MMP), metabolic rewiring (glucose transporter 1, GLUT1), pH homeostasis (carbonic anhydrases, CAs), drug efflux (multidrug resistance 1, MDR1). These coordinated transcriptional programs promote the selection of tumor clones adapted to survive in unfavorable conditions, characterized by chemoresistant and pro-metastatic phenotypes

Given the high heterogeneity of TME in terms of O_2_ and nutrients supply, cancer cells must continuously adapt to conditions that change spatially and temporally. If the process fails, pro-apoptotic pathways prevail and cells are eliminated. If the process is successful, this continuous adaptation promotes cell survival and tumor progression. The first scenario sensitizes cells to the damages exerted by radiotherapy and chemotherapy; the second scenario is unequivocally associated to therapy-resistance (Fig. 1).

## Hypoxia and altered UPR: two partners in crime of chemoresistance

### Hypoxia drives the acquisition of chemoresistance

In vitro, hypoxia has been reported to induce resistance to several anti-cancer drugs, including Vinka alkaloids, anthracyclines, cisplatin, etoposide, actinomycin-D, 5-fluorouracil, gemcitabine, methotrexate, in different solid cancers [90].

Both HIF-1α-dependent and indepedent mechanisms are responsible for the chemoresistance related to hypoxia. It is well documented that hypoxic tumors have a poor vascularization that limits drug bioavailability [91]. In addition, the low O_2_ tension reduces the possibility of inducing oxidative stress that is often a mechanism of killing exerted by chemotherapeutic drugs [36], such as cisplatin [92], doxorubicin, etoposide [93], gemcitabine [94]. Moreover, by creating an unfavorable environment, hypoxia selects aggressive and metastatic clones, as well as clones rich of anti-apoptotic proteins such as IAP3 and Bcl-2: this selection is independent on HIF-1ɑ activation [95], but it contributes to counteract the apoptosis induced by anti-cancer agents.

**Fig. 2:**
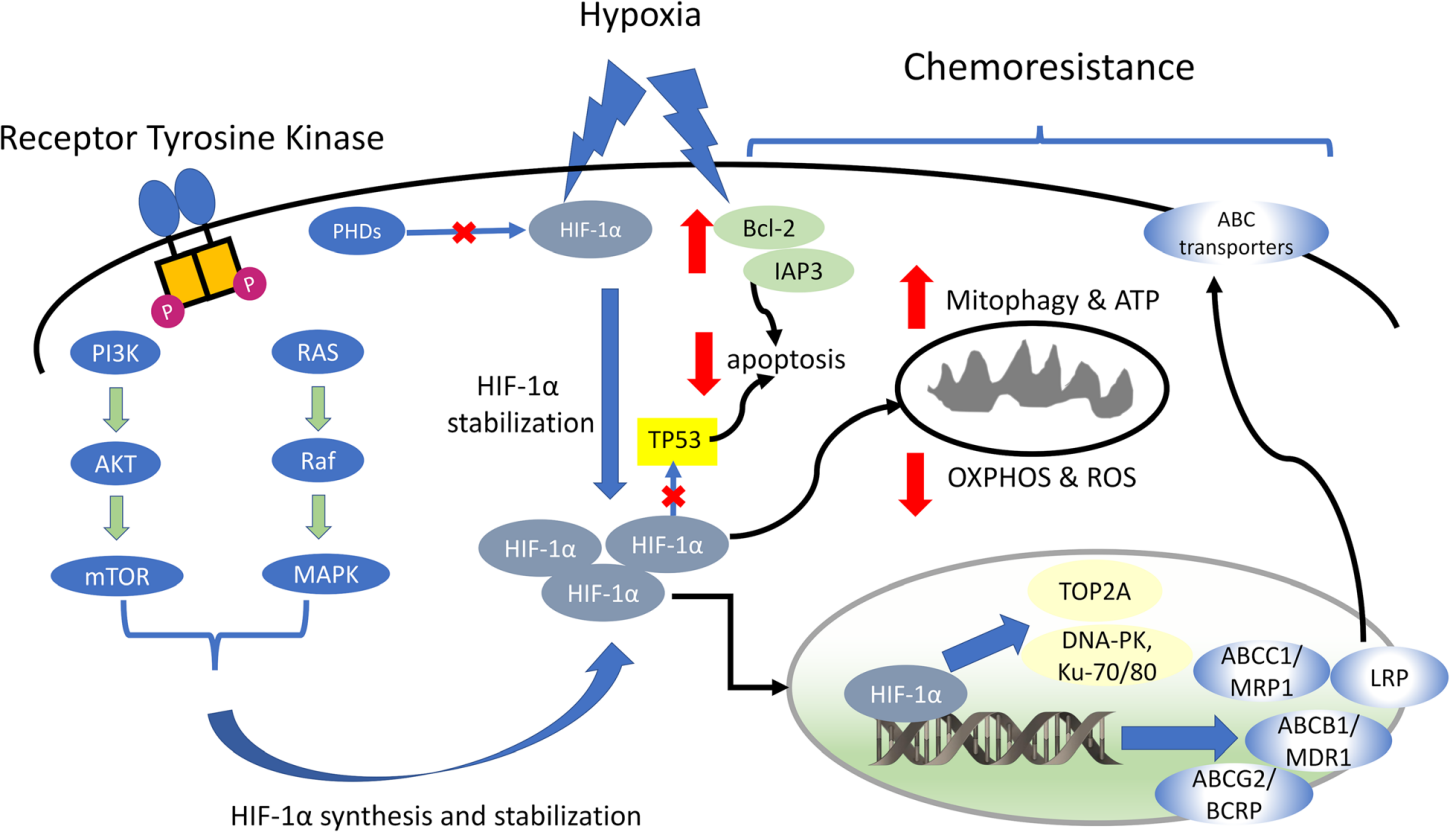
Hypoxia induces chemoresistance. Besides the stabilization of the hypoxia-inducible factor-1α (HIF-1α) due to the low activity of prolyl hydroxylase dioxygenase (PHDs) enzymes, oncogenically activated axes downstream receptor tyrosine kinases, such as PI3K/Akt/mTOR and Ras/Raf/MAPK axes, contribute to increase the amount of transcriptionally active HIF-1α. Among the target genes there are several ATP binding cassette (ABC) transporters involved in chemotherapeutic drug efflux (e.g. ABC transporter B1/multidrug resistance 1, ABCB1/MDR1, encoding for P-glycoprotein; ABC transporter C1/multidrug drug resistance related protein 1, ABCC1/MRP1; ABC transporter G2/breast cancer resistance protein, ABCG2/BCRP; lung resistance protein, LRP), and genes involved in DNA repair, such as topoisomerase 2A (TOP2A), DNA-PK, Ku-70 and Ku-80, preventing the DNA damage elicited by chemotherapy. HIF-1α also inhibits TP53-induced apoptosis in repsonse to chemotherapy, by destabilizing TP53. In addition, hypoxia is associated with other events determining chemoresistance, such as the increase in mitophagy that spares ATP, the reduction of oxidative phosphorylation (OXPHOS) and reactive oxygen species (ROS) that reduce oxidative damage, the increase of the anti-apoptotic proteins Bcl-2 and IAP3. The sum of these hypoxia-driven responses, either HIF-1α-dependent or independent, make hypoxic cells highly chemoresistant

Most frequently, the ample transcriptional program induced by HIF-1α is the main responsible for the simultaneous resistance to drugs unrelated for structures and mechanisms of action (Fig. 2). Among the main target of HIF-1α there is *mdr1* gene, which encodes for P-glycoprotein/ATP binding cassette transporter B1 (Pgp/ABCB1) [96]. This protein effluxes many chemotherapeutic drugs, contributing to tumor multidrug resistance (MDR) [97]. In vitro 3D-models mimic well the chemoresistance observed in the hypoxic core of solid tumors: both hormone-dependent and TNBC 3D models display a significant activation of HIF-1α, coupled with an increased Pgp up-regulation and resistance to doxorubicin, compared to parental 2D-cultured doxorubicin-sensitive cells [98, 99]. The reversion of doxorubicin resistance and the down-regulation of Pgp after HIF-1α inhibition with 3-(5′-hydroxymethyl-2′-furyl)-1-benzimidazole or HIF-1α shRNA, prove the molecular linkage between hypoxia and HIF-1α-driven Pgp expression [98]. Notably, chemoresistant tumors often have activated HIF-1α in normoxia, as a result of the activation of Ras/ERK1/2 and RhoA/RhoA kinase axes that phosphorylate and stabilize HIF-1α. This kind of HIF-1α activity was detected in colon cancer, malignant pleural mesothelioma, non-small cell lung cancer (NSCLC), TNBC and chronic lymphatic leukemia cells [100–104]. This series of studies highlights that HIF-1α alone is sufficient to induce a MDR phenotype by increasing the expression of Pgp, independently of hypoxia. In line with this observation, the oncogenic-driven activation of HIF-1α is as strong as the hypoxic TME in determining chemoresistance. Hence, we may hypothesize that HIF-1α-dependent chemoresistance occurs both in hypoxic tumor areas and in well-oxygenated ones characterized by a hypoxia-independent activation of HIF-1α. These two scenarios are not antagonistic, but can co-exist, as in the case of multiple myeloma (MM): growing in an hypoxic niche in bone marrow, MM has a canonical activation of HIF-1α that induces chemoresistance, but also the concurrent activation of oncogenic pathways (e.g. Wnt, Notch, Ras/MAPK-, PI3K, Akt/mTOR-, NF-kB-dependent pathways) that prevent the apoptosis induced by chemotherapeutic drugs [90, 105].

Other MDR-related transporters are under the transcriptional control of HIF-1α, such as MDR related protein 1/ABC transporter C1 (MRP1/ABCC1) and lung resistance protein (LRP) [106]. Breast cancer resistance protein/ABC transporter G2 (BCRP/ABCG2) is up-regulated by both HIF-1α [107], which is phosphorylated and stabilized by ERK1/2 [108], and HIF-2α [109]. The simultaneous up-regulation of different transporters enormously enlarges the spectrum of drugs that lose efficacy in hypoxia or in tumors with a constitutively active HIF-1α.

An additional tumor-intrinsic mechanism linking hypoxia to chemoresistance involves the HIF-1α-driven inhibition of TP53. Both HIF-1α and HIF-2α suppress TP53-mediated apoptosis. This activity has been linked to the resistance to 5-fluorouracil in TP53 wild-type gastric cancers, but not in TP53-disrupted ones [110]. Notably, on the one hand HIF-1α destabilizes TP53, on the other hand the binding of TP53 to HIF-1α impairs the hypoxia-driven transcriptional program [111]: depending on the mutational status of TP53 and/or on the amount of HIF-1α, the interaction between these two partners may produce pro-survival effects or cell death. These two extremities oscillate from chemoresistance in the first case to chemosensitivity in the second case.

HIF-1ɑ also prevents DNA double strand breaks, e.g. by up-regulating topoisomerase 2A. This mechanism confers resistance to etoposide in TNBC and PC [112]. This observation leads to the use of dual HIF/topoisomerase inhibitors (such as acriflavine) as potential anticancer and chemosensitizer agents [113]. At the same time, HIF-1ɑ increases the expression of DNA repair machinery, such as DNA-PKs, Ku80 and Ku70 [114]: this mechanism, which contributes to the adaptation and survival in conditions of chronic hypoxia [115], also protects from DNA-damaging chemotherapeutic drugs.

The metabolic rewiring driven by hypoxia also plays a role in chemoresistance [116]. The huge production of lactate due to the increase in glycolysis determines a strong acidification of the TME that limits the efficacy of weak bases such as anthracyclines: indeed, these drugs are easily protonated and sequestered within lysosomes [117]. In the attempt of counteracting the strong acidification, cancer cells up-regulate carbonic anhydrase (CA) IX and XII, both under the transcriptional control of HIF-1α [118, 119]. Also this event, however, is functional to induce chemoresistance. Indeed, CAXII interacts with Pgp and stimulates its activity, by creating a slightly alkaline pH in the membrane domains where the protein operates [119], and granting the maximal efficiency of the pump [120].

By down-regulating OXPHOS [30] and inducing mitophagy [121, 122], HIF-1α favors chemoresistance by multiple and interconnected mechanisms. Chemotherapeutic drugs as 5-fluorouracil and cisplatin increase ROS generation [110]. The lower levels of OXPHOS detected in hypoxic cells or in cells with high levels of HIF-1α may explain the lower generation of ROS and the reduced oxidative damages induced by chemotherapy in these conditions [123]. Moreover, the reductive catabolism of glutamine – i.e. the cytosolic transformation of glutamine into citrate – is a hallmark of hypoxic tumors [124]. This pathway produces NADPH, provinding additional ROS-buffering agents that limit the oxidative damages elicited by chemotherapy. Of note, ROS are also necessary to stabilize TP53 and trigger apoptosis [110]: therein, HIF-1α-positive cells have a lower pro-apoptotic activity of TP53 in response to chemotherapeutic drugs as cisplatin [125]. HIF-2α exerts a similar contribution by decreasing intracellular ROS and limiting the stability of TP53 [110], thus providing an additional loop that induces resistance to oxidative stress and chemotherapy. Mitophagy allows cancer cells to recover ATP, redox metabolites and building blocks, that can be used to re-synthesize biomolecules damaged by chemotherapeutic drugs, providing an additional mechanism by which the altered mitochondrial metabolism induced by HIF-1α determines chemoresistance. For instance, HIF-1α triggers a protective mitophagic response and at the same time confers resistance to 5-fluorouracil [126], gemcitabine [94] and cisplatin in ovarian cancer [123]. Indeed the increased rate of mitophagy [121, 122], favored by the up-regulation of the mitophagic Bcl-2/adenovirus E1B 19-kDa interacting protein 3 (BNIP3) by HIF-1α [127], compensates the lower ATP produced by OXPHOS. ATP is a necessary substrate for ABC transporters and favors the efflux of multiple chemotherapeutic drugs.

HIF-1α is also a strong inducer of glutaminolysis [128] that can be reduced into citrate and generate NADPH [124], or can fuel the TCA cycle. This anaplerotic pathway, together with the reshaping of mitochondrial cristae that maximizes the ATP synthesis [129] and the increased mitobiogenesis caused by the up-regulation of the peroxisome proliferator-activated receptor gamma coactivator-1α (PGC-1α) observed in hypoxic and acidic tumors [130], compensate the attenuated TCA cycle and OXPHOS induced by hypoxia.

This metabolic phenotype allows hypoxic cancer cells to rely mainly on a glycolytic metabolism, but to switch toward a mitochondrial metabolism when glucose is low. This plasticity ensures a high adaptability to the metabolic changing conditions of the TME, granting the possibility of supplying energy and building blocks by rewiring the metabolism. As a consequence, hypoxic cells are less susceptible to the oxidative and energetic damages produced by chemotherapy.

Other chemoresistance inducers that can be up-regulated by HIF-1α are Pim kinases members [131] which phosphorylate and activate Pgp [132]. Also the NF-kB and Akt/mTOR axes, which are increased in hypoxia, may activate Pim kinases [121], amplifying chemoresistance. Collectively, Pim kinases have been implicated in resistance to cisplatin, doxorubicin and gemcitabine in pancreatic adenocarcinoma (PDAC) cells, and to docetaxel in PC cells [132, 133]. Interestingly, also specific miRNAs associated with HIF-1α up-regulation are involved in chemoresistance. For instance, the HIF-1α/mir-210 axis determines resistance to temozolomide in glioblastoma by increasing the cell proliferation and the acquisition of a stemness phenotype [134]. In keeping with these results, mir-210 is considered a biomarker predictive of chemoresistance in patients with metastatic breast cancer [135]. Mir-519c up-regulates ABCG2 [136], a gene target of HIF-1α [107], providing an explanation for the ABCG2-mediated chemoresistance detected in tumors with high expression of HIF-1α. mir-10b confers resistance to 5-fluorouracil in colorectal cancer, where it inhibits the pro-apoptotic BH3-only Bcl-2 family member BIM (BCL2L11) and is considered a negative prognostic factor in chemotherapy-treated patients [137]. Similarly, miR-155, which determines chemoresistance in solid and hematological tumors [138], is considered a negative prognostic marker in breast cancers [139]. The correlation between high HIF-1α, high levels of miRNAs dependent on HIF-1α and chemoresistance, however, is not univocal. Indeed, mir-372 is a predictor of chemosensitivity in colorectal cancer [140] and mir-373 chemosensitizes gastric cancer cells [141], although they are both increased by HIF-1α [24]. Thse data suggest that many other factors can intervene to determine if a specific miRNA induced by hypoxia causes chemoresistance or not.

Senescence is another process often associated with drug resistance [142] and sustained by HIF-1α, both in normoxia [110] and hypoxia [143]. Hypoxic cells are often quiescent or slowly cycling [91, 144]. These types of cells are the most difficult to eradicate and the most resistant to drugs interfering with cell cycle, such as pemetrexed and raltitrexed [145]. By contrast, the sensitivity towards drugs acting in a cell cycle-independent manner, such as proteasome inhibitors, is preserved [145].

Finally, HIF-1α increases resistance to chemotherapy by promoting EMT [36], which induces cell proliferation and migration, as demontstrated by the down-regulation of E-cadherin and the up-regulation of vimentin in hypoxic PC cells. Propofol, recently identified as a HIF-1α inhibitor, not only reduces EMT but also sensitizes cells to docetaxel [146]. This mechanism is not drug- or tumor-type specific, because it is elicited also by the natural product tanshinone IIA, another HIF-1α inhibitor that impairs EMT and alleviates the hypoxia-induced resistance to doxorubicin in breast cancer cells [147].

In general, hypoxic tumors display multiple and redundant mechanisms of resistance to chemotherapy. Some molecular circuitries involved in chemoresistance are part of the adaptation strategies to hypoxia or unfavorable conditions. If we consider chemotherapy as one unfavorable condition that cancer cells encounter, we might hypothesize that cells adapted to survive in hypoxia should be more prone to survive under the selective pressure of chemotherapy. An accurate choice of the type of chemotherapeutic drugs, together with the combination of HIF-1α inhibitors, may ameliorate the response of hypoxic tumors to chemotherapy.

### An altered UPR impacts on sensitivity to chemotherapy

Few evidences reported that ER stress induces chemosensitivity, as in the case of ovarian cancer cells and cisplatin [148], or gastric cancer and doxorubicin/vincristine [149], but in both cases the higher chemosensitivity seems independent from the resistance to ER stress. Since under ER stressing conditions cells are depleted of ATP if they cannot recover it via autophagy, they activate a UPR-dependent apoptosis. At the same time, the low levels of ATP deprive Pgp and other ABC transporters of the energetic substrate for pumping out chemotherapeutic drugs. These mechanisms may explain why Pgp-expressing ovarian cancer cells subjected to ER stress are sensitizied to paclitaxel [150].

Most often, however, an increased resistance to ER stress, as it occurs in cells adapted to survive in a hypoxic environment, determines chemoresistance.

For instance, glucose deprivation activates GRP78 that in turn increases *mdr1* gene transcription via c/Jun [151] and mounts a cytoprotective autophagic reaction in response to bortezomib in MM [152] or to vemurafemib in resistant BRAF^V600E^ mutated melanoma cells [153]. On the contrary, betulinic acid-induced activation of GRP78 increases PERK/CHOP axis, promoting the apoptosis induced by taxol [154]. In accordance, the down-regulation of GRP78, coupled with the up-regulation of IRE1α, is associated with resistance to cisplatin in NSCLC cells [155]. Also GRP78 downstream effectors may induce chemoresistance. In PDAC cells, PERK induces chemoresistance by phosphorylating eIF2α that attenuates protein translation and prevents the accumulation of unfolded proteins within ER lumen, thus alleviating the proteostatic stress. At the same time PERK activates ATF4/CHOP axis that - contrarily to most cell types where CHOP is a pro-apoptotic factor [156] - exerts anti-apoptotic effects and induces resistance to gemcitabine [157]. The resistance to gemcitabine in PDAC stem cells has been also linked to the down-regulation of IRE1α and the simultaneous activation of PERK, that in turn up-regulate the urokinase plasminogen activator (uPA). uPA prevents the mitochondria-dependent apoptosis elicited by gemcitabine [158], thus promoting chemoresistance by activating a cross talk between ER and mitochondria. Defective ATF6 and XBP-1 determine resistance to bortezomib in MM, coupled with reduced ER lumen and reduced ability to mount a UPR-triggered cell death in response to proteostatic stress [159]. Accordingly, the block of E1-ubiquitin-activating enzyme and the consequent proteostatic stress increase the expression of IRE1α, PERK and ATF6 that cooperate in inducing apoptosis and increasing sensitivity to proteasome inhibitors, doxorubicin, melphalan and lenalidomide [160].

As it occurs for the chemoresistance associated with HIF-1α, the presence of oncogenic drivers pushes the activation of specific ER stress sensors that induce chemoresistance. For instance, in chronic myeloid leukemia cells the BCR/ABL oncogene determines the constitutive phosphorylation of PERK and eIF2α that prevent the ER stress-dependent apoptosis in response to imatinib, as demonstrated by the re-sensitization to the drug when PERK/eIF2α activity is prevented [161]. In colon cancer, breast cancer and osteosarcoma, PERK determines resistance to oxaliplatin and doxorubicin, via the activation of erythroid-derived 2-like 2 (Nrf2), which up-regulates anti-oxidant enzymes and MRP1 [99], two actors in the chemoresistant phenotype.

In a strict parallelism with the linkage between hypoxia and chemoresistance, also the linkage between resistance to ER stress and resistance to chemotherapy is reciprocal. On the one hand, cells selected to survive under mild and chronic ER stress conditions acquire drug resistance by up-regulating PERK, similarly to cells selected in medium with increasing concentrations of chemotherapeutic drugs that acquire a MDR phenotype [95]. On the other hand, cells with a MDR phenotype are also resistant to ER stress induced-cell death: both ER stressors and chemotherapeutic drugs – such as doxorubicin, oxaliplatin/cisplatin, 5-fluorouracil – increase the CCAAT/enhancer-binding protein-β (C/EBP-β) LIP isoform that prevents the CHOP/caspase 3-mediated apoptosis and at the same time up-regulates Pgp [162]. Another linkage between ER stress adaptation/resistance and chemoresistance is provided by the observation that chemoresistant tumors often exhibit a defective ERAD/ERQC, making them constantly subjected to a chronic proteostatic stress. The adaptation to this chronic stressing condition constitutively activates ER-dependent pro-survival pathways that contribute to chemoresistance [163, 164]. However, perturbing the unstable balance between defective ERAD/ERQC system and compensatory up-regulation of pro-survival pathways may hit ER stress-resistant/chemoresistant cells in their Achille’s heel. Indeed, inducing ER stress with ER-targeting doxorubicin perturbing disulfide bonds of proteins [164] or Ag-nanoparticles inducing ER stress [165] restore chemosensitivity. The mechanism is double. First, the accumulation of misfolded proteins induces their ubiquitination and the engulfment of proteasome/autophagosome system. Second, Pgp is folded within ER and stabilized by disulfide bonds: the interference with these processes promotes its degradation, with consequent decreased efflux of chemotherapeutic drugs and sensitization to Pgp substrates [164–166].

Overall, the different stimuli that specifically activate one or more UPR arms, the different nature and timing of ER stressing conditions, the different pattern of UPR downstream transducers explain why the mechanisms linking ER stress and chemoresistance are multiple and sometimes contrasting in different tumors. This variable scenario is further complicated in the core of solid tumors, because hypoxia is a cause of both ER stress and chemoresistance, and may trigger additional circuitries increasing at the same time the resistance to ER stressing conditions and to chemotherapeutic drugs. For these reasons, hypoxic tumors should be considered characterized by a “multi-resistant” phenotype.

### The crosstalk between hypoxia and UPR-dependent circuitries induces chemoresistance

**Fig. 3:**
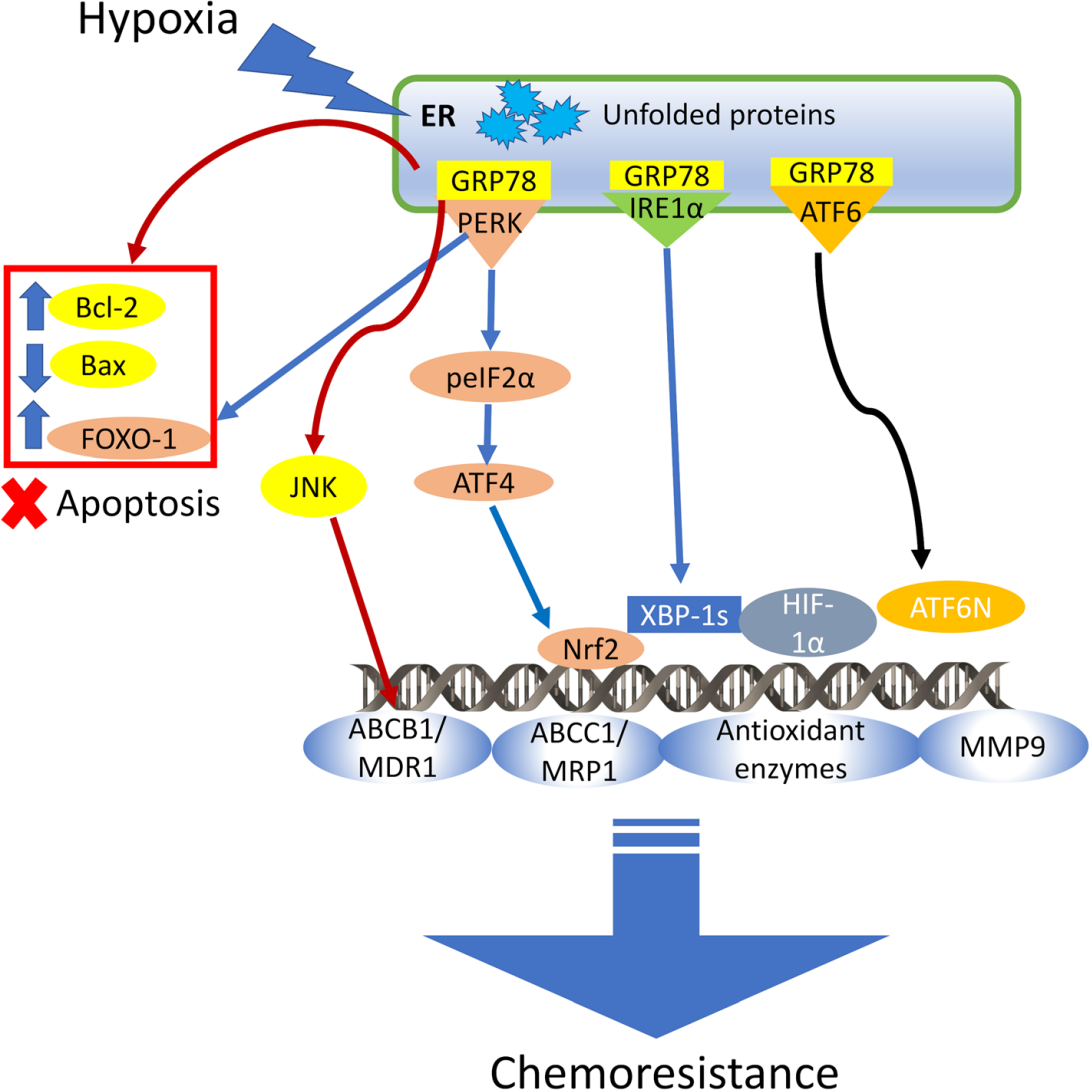
Hypoxia and UPR cooperate in inducing chemoresistance. In hypoxic cells, the ER stress sensors are activated and cooperate in inducing chemoresistance. glucose-regulated protein 78 (GRP78) increases the anti-apoptotic Bcl-2/Bax ratio and transcriptionally induces ABC transporter B1/multidrug resistance 1 (ABCB1/MDR1) gene by activating Janus kinase (JNK). Protein kinase R-like endoplasmic reticulum kinase/eukariotic initiating factor 2α/activating transcription factor-4 (PERK/eIF2α/ATF-4) axis stabilizes the anti-apoptotic factor forkhead box O-1 (FOXO-1) and activates the transcription factor erythroid-derived 2-like 2 (Nrf2), which in turn up-regulates ABC transporter C1/multidrug resistance related protein 1 (ABCC1/MRP1), antioxidant enzymes and matrix metalloprotease 9 (MMP9). Together with PERK-dependent signalling, also inositol-requiring enzyme-1α/X-box-binding protein 1 (IRE1α/XBP-1) and activating transcription factor-6 (ATF6)-dependent axes support hypoxia-inducible factor-1α (HIF-1α) transcriptional program, contributing to the chemoresistance typical of hypoxic tumors

Being two hallmarks of resiliency in cancers, hypoxia and altered UPR response can cooperate in inducing chemoresistance. Multiple crosstalks exist between the pro-survival pathways activated by HIF-1α and UPR (Fig. 3), and they can be enhanced by additional pro-oncogenic conditions, such as mutated TP53 [167].

Hypoxia increases the expression of GRP78 that in turn promotes an adaptive UPR response to the hypoxic environment: peculiar events in this adaptation are the down-regulation of CHOP and Bax, and the up-regulation of Bcl-2. The increased Bcl-2/Bax ratio results in the inhibition of apoptosis; in this way, the hypoxic-driven up-regulation of GRP78 triggers resistance to cisplatin [168]. Accordingly, GRP78-silencing restores chemosensitivity, rewiring the pattern of CHOP/Bax/Bcl-2 expression, notwithstanding the persistence of an hypoxic environment [168]. GRP78 [151], as well as HIF-1α [96], are transcriptional inducers of *mdr1*. Therefore, hypoxia inevitably leads to resistance towards the multiple chemotherapeutic drugs transported by Pgp. The accumulation of GRP78 and prolyl 4-hydroxlase, beta polypeptide (P4HB) in the ER lumen of hypoxic glioblastoma cells maintains low the activity of IRE1α and PERK/CHOP. Hyperoxia not only restores the relative levels of PERK/CHOP and IRE1α, by reducing GRP78 and P4HB, but it also enhances the apoptosis induced by temozolomide [169]. Although temozolomide mainly induces cell death by alkylating DNA, in an ER-independent way, the sensitization to the drug cytotoxicity is likely due to the induction of “two apoptotic hits”, the first one at the DNA level, the second one at the ER level, as suggested by the synergism achieved by hyperoxia and temozolomide.

PERK/eIF2α/ATF4 axis is typically induced by hypoxia [56] and contributes to the hypoxia-induced chemoresistance with multiple mechanisms. First, PERK increases the phosphorylation and activity of the anti-apoptotic forkhead box O-1 (FOXO-1), together with pro-autophagic factors [170]. The inhibition of apoptosis and the increased autophagy concurrently protect cells from chemotherapeutic drugs. Again, UPR-dependent and hypoxia-dependent axes synergize, because HIF-1α induces FOXO-1 transcription [171], promotes protective autophagy [172] and activates PERK/eIF2α/ATF4 axis [56]. By cooperating with HIF-1α in up-regulating MMP9 [173], PERK and HIF-1α also promote migration, helping cancer cells to leave unfavorable environments and escape from cytotoxic agents. As mentioned above, Nrf2 is also activated by PERK and confers chemoresistance by up-regulating MRP1 [99] and anti-oxidant enzymes [170]. Of note, hypoxia-associated ROS up-regulate both HIF-1α and Nrf2: the sum of their transcriptional programs unequivocally worsens chemoresistance [174].

IRE1α/XBP-1 [79] and ATF6 [175] are activated in hypoxic cells as well, although the role of such increase in chemoresistance is not univocal. While the increase of ATF6 and the decrease of IRE1α has been linked to resistance to cisplatin in NSCLC [155], in MM the low activity of both ATF6 and IRE-1α induces resistance to bortezomib [159]. By contrast, XBP-1 up-regulation determines a more aggressive phenotype in TNBC. In this tumor, XBP-1 gene signature is strongly associated with HIF-1α gene signature, and they correlate with a lower relapse-free survival [84]. Since chemotherapy is the first treatment option for TNBC, the high HIF-1α/high XBP-1 phenotype could be indicative of poor response to chemotherapeutic drugs and consequent lower survival.

In addition, hypoxic niches are enriched of cancer stem cells (CSCs) that are usually chemoresistant [176], because they have higher expression of ABC transporters [177, 178] and HIF-1α-mediated up-regulation of MMP9, C-X-C chemokine receptor type 4 (CXCR4), osteopontin, IL-8 and VEGF [179] that promote invasion and chemoresistance. In addition, CSCs may show a differential UPR response than differentiated cells [180]. For instance, cervical CSCs show high resistance to the ER stress cell death induced by tunicamycin, because they down-regulate pro-apoptotic pathways dependent from IRE1α and activate pro-survival pathways dependent from PERK. This shift also induces cisplatin resistance that can be reversed by specific inhibitors of IRE1α and PERK [180]. Colon CSCs have down-regulated specific mediators of ER stress-dependent cell death (such as CHOP) and are more resistant to chemotherapy with oxaliplatin, 5-fluorouracil and irinotecan. Inducing ER stress with subtilase cytotoxin A, that cleaves and activates GRP78, promotes chemosensitization and loss of stemness phenotype [181]. Paradoxically, chemotherapeutic drugs that induce a pro-apoptotic UPR in differentiated cells, such as gemcitabine, activate pro-survival and pro-invasive pathways dependent on ATF6 in PDAC CSCs [158]. These findings suggest that the acquisition of stemness properties, driven by the growth in hypoxic environment, induces an altered UPR that mediates chemoresistance.

The experimental evidences reported above clearly indicate that the mechanisms linking UPR, hypoxia and chemoresistance are multiple and often tumor-specific. Being the results of an adaptive program to unfavorable conditions, these mechanisms must be considered in a dynamic perspective. Depending on the degree of hypoxia, timing and exposure to chemotherapeutic drugs, the same molecular axis can be turned-on or turned-off, activating opposite and compensatory pathways that lead to cell death or survival.

## Conclusions and future perspectives

**Fig. 4:**
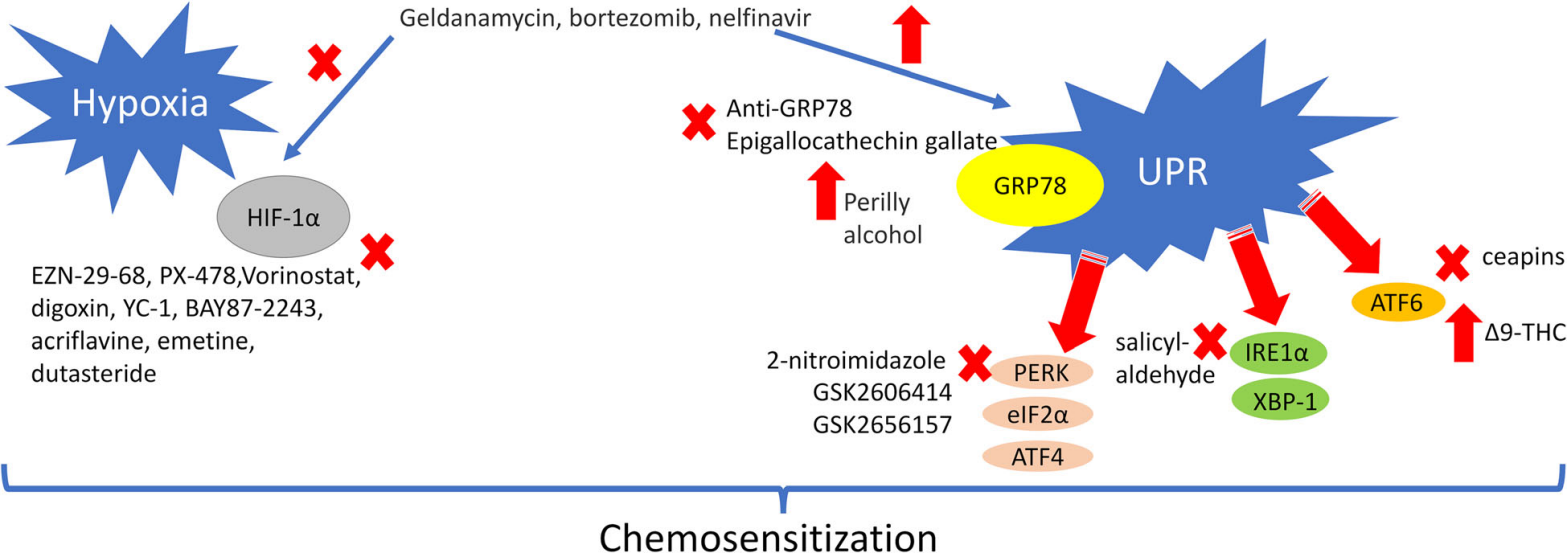
HIF-1α- and UPR-targeting drugs as new chemosensitizing agents. Pharmacological inhbitors (*red crosses*) or activators (*red arrows*) of hypoxia-inducible factor-1α (HIF-1α) or unfolded protein response (UPR) actors - glucose-regulated protein 78 (GRP78), protein kinase R-like endoplasmic reticulum kinase/eukariotic initiating factor 2α/activating transcription factor-4 (PERK/eIF2α/ATF-4), inositol-requiring enzyme-1α/X-box-binding protein 1 (IRE1α/XBP-1) and activating transcription factor-6 (ATF6) – can be repurposed as chemosensitizing agents in hypoxic tumors. Δ9-THC: Δ9-tetrahydrocannabinol

In solid tumors the hypoxic microenvironment determines a continuous ER stress. Like in the aphorism “What does not kill me, makes me stronger”, the combined pressure of two unfavourable conditions eliminates the most sensitive cells, but it trains the most resistant cells to survive and mount a network of adaptive responses to environmental stresses. This ability is plastic and determines the progressive acquisition of multiple resistances, e.g. resistance to oxidative stress, to nutrient shortage, to chemotherapy. As a result of this selective pressure, the most chemoresistant and aggressive clones emerge during tumor progression. Since hypoxia induces pro-survival UPR-dependent pathways and chemoresistance, and an altered UPR mediates chemoresistance both in normoxia and hypoxia, disrupting these vicious circles may help in finding new chemosensitizing strategies or drug combinations inducing synthetic lethality (Fig. 4).

Several UPR activators (e.g. GRP78 activator perilly alcohol, ATF6 activator Δ9-tetrahydrocannabinol) or inhibitors (e.g. GRP78 blocker epigallochetchin gallate, ATF6 blocker ceapins, IRE1-α inhibitors salicylaldehyde-derivatives, PERK inhibitors GSK2606414 and GSK2656157) have been employed as inducers of apoptosis in cancer preclinical models [182–184], but only few of them have reached clinical trials (https://clinicaltrials.gov/ct2/results?term=drug+targeting+UPR). The multiple crosstalks, as well as the involvement of UPR in several physiologic processes in non-transformed tissues, make it difficult to have safe and effective therapeutic strategies at the present. For instance, GSK2656157 is a direct inhibitor of PERK: since PERK is a key actor in the hypoxia-mediated chemoresistance, it has been administered in PDAC xenografts that are notoriously refractory to the majority of treatments [185]. Although GSK2656157 successfully reduced tumor growth, decreased vessel density and altered aminoacid metabolism, it also damaged pancreatic β-cells [185]. Therefore, the risk of insulin resistance or diabetes disencouraged from using GSK2656157 in patients. To limit the damages on normal tissues, hypoxia-activated PERK inhibitor prodrugs have been produced, by using a 2-nitroimidazole moiety that is enzymatically reduced in hypoxic conditions into the active drug. The first tests in colon cancer cells were promising, although these prodrugs effectively inhibited PERK only in the micromolar range [186]. At this concentration, the risk of off-target effects is high, raising questions on the feasibility of this approach in patients.

Also, HIF-1α targeting is complicated because of the multiple pathways that control HIF-1α expression and of the multiple transcriptional programs that are controlled by HIF-1α. Inhibitors of HIF-1α translation (EZN-29-68, PX-478, Vorinostat, digoxin), stability (PX-478, Vorinostat, YC-1) and transcriptional activity (YC-1, acriflavine), or inhibitors of HIF-1α upstream inducers, as PI3K/Akt/mTOR axis, are the most used drugs at the present [187, 188]. Some of these compounds have shown good chemosensitizing effects. In several preclinical models of solid and hematologic tumors, direct inhibitors of HIF-1α, such as YC-1 [103] and BAY87-2243 [189], or indirect down-regulators of HIF-1α signalling, as the anti-androgen dutasteride [190], the alkaloid emetine [191], the lncRNA HITT [192] – one of the multiple ncRNA that regulate HIF-1α [193] –, have induced chemosensitization, by reducing HIF-1α transcriptional activity. The disadvantage of these inhibitors is their aspecificity. In order to overcome this limitation, the first liposomal formulations containing acriflavine co-encapsulated with doxorubicin have been produced, and demostrated a superior tumor targeting and anti-tumor efficacy than doxorubicin alone [173]. Currently, more than 100 trials are testing HIF-1α inhibitors as anti-cancer agents (https://clinicaltrials.gov/ct2/results/details?cond=Cancer&term=HIF), despite the results were below the expectations. A partial explanation of this failure is that HIF-1α is a hallmark of aggressive and resistant cancers. Indeed, HIF-1α presence is a synonimous of cells characterized by multiple and redundant resistance pathways: the inhibition of HIF-1α can only partially turn-off their pro-survival attitude.

Pharmacological interventions on multiple pathways can achieve better results. In this perspective, the simultaneous targeting of UPR and HIF-1α can be considered a valid approach. For instance, geldanamycin, a HSP90 inhibitor, induces at the same time ER stress and HIF-1α ubiquitination, down-regulating its angiogenetic transcriptional program [194]. Bortezomib and nelfinavir, two drugs able to elicit ER stress by inducing a proteostatic stress [159, 195], also down-regulate HIF-1α activity [196, 197]. These few examples highlight that the dual targeting of UPR and HIF-1α with FDA-approved drugs is feasible. This drug-repurposing strategy can be exploited as a new chemosensitizing approach, although problematic toxicities in patients cannot be excluded also in this case.

Although not translable to patients in the present, the pharmacological and mechanistic studies on the cross-talk between hypoxia, UPR and chemoresistance, are useful to put together the pieces of the puzzle linking these three players. Increasing our understanding on these circuitries will steer the pharmacological research towards more precise and effetive approaches to counteract hypoxic and chemoresistant tumors in the next future.

## Data Availability

Not applicable.

## Abbreviations

ATF4: activating transcription factor-4 (ATF4)
ATF6: activating transcription factor-6
BCRP/ABCG2: breast cancer resistance protein/ATP binding cassette transporter G2
CAFs: cancer associated fibroblasts
C/EBP-β LIP: CCAAT/enhancer-binding protein-β LIP
CEMIP: cell migration-inducing and hyaluronan-binding protein
CHOP: CCAAT/Enhancer-binding protein homologous protein
CPT1A: carnitine palmitoyl transferase 1A
ECM: extracellular matrix
eIF2α: eukariotic initiating factor 2α
EMT: epithelial-mesenchymal transition
ER: endoplasmic reticulum
ERAD/ERQC: ER-associated protein degradation/ER-quality control system
ERK: extracellular signal-regulated kinase
ETC: electron transport chain
FAO: fatty acid β-oxidation
FOXO-1: forkhead box O-1
GLUT1: glucose transporter 1
GRP78: glucose-regulated protein 78
HCC: hepatocellular carcinoma
HEY: Hes-related family BHLH transcription factor with YRPW motif repressor
HGF: heptocyte growth factor
HIF: hypoxia-inducible factor
IRE1α: inositol-requiring enzyme-1α
JNK1: c-Jun N-terminal kinase
LAMP: lysosomal-associated membrane protein 3
LRP: lung resistance protein
MAPK: p38 mitogen activated kinase
MDR: multidrug resistance
MM: multiple myeloma
MMP9: matrix metalloprotease 9
MRP1/ABCC1: multidrug resistance related protein 1/ATP binding cassette transporter C1
mTOR: mammalian target of rapamycin
mUPR: mitochondrial unfolded protein response
Nrf2: nuclear factor erythroid-derived 2-like 2
NSCLC: non-small cell lung cancer
O_2_: oxygen
OXPHOS: oxidative phosphorylation
PC: prostate cancer
PDAC: pancreatic adenocarcinoma
PERK: protein kinase R-like endoplasmic reticulum kinase
Pgp/ABCB1: P-glycoprotein/ATP binding cassette transporter B1
PHDs: prolyl hydroxylase dioxygenase enzymes
PKC: protein kinase C
PLC: phospholipase C
pVHL: von Hippel Lindau tumor suppressor protein
ROS: reactive oxygene species
TAM: tumor-associated macrophage
TCA: tricarboxylic acid cycle
TH17: T-helper 17 cells
TME: tumor microenvironment
TNBC: triple negative breast cancer
TOPA2A: topoisomerase 2A;
uPA: urokinase plasminogen activator
UPR: unfolded protein response
VEGF: vascular endothelial growth factor
VEGFR: vascular endothelial growth factor receptor
XBP-1: X-box-binding protein 1
